# Decisional conflict and knowledge in women with *BRCA1/2* pathogenic variants: An exploratory age group analysis of a randomised controlled decision aid trial

**DOI:** 10.1371/journal.pone.0311432

**Published:** 2024-10-24

**Authors:** Sibylle Kautz-Freimuth, Zoë Lautz, Arim Shukri, Marcus Redaèlli, Kerstin Rhiem, Rita Schmutzler, Stephanie Stock

**Affiliations:** 1 Institute of Health Economics and Clinical Epidemiology, Faculty of Medicine and University Hospital Cologne, University of Cologne, Cologne, Germany; 2 Centre for Hereditary Breast and Ovarian Cancer and Centre for Integrated Oncology (CIO), Faculty and University Hospital Cologne, University of Cologne, Cologne, Germany; CNR, ITALY

## Abstract

Female *BRCA1/2* pathogenic variant (PV) carriers face substantial risks for breast and ovarian cancer. Evidence-based decision aids (DAs) can facilitate these women in their decision-making process on an individually suitable preventive strategy. However, there is a gap in previous literature exploring whether DA effectiveness varies according to women’s age. This is an exploratory subanalysis with a descriptive approach from a randomised controlled study assessing the effectiveness of a German decision aid (DA) for women with *BRCA1/2* PVs compared to no DA use. From the original sample, women aged 18–40 years and >40 years and the intervention and control groups (IG, CG) within each of the age groups were compared regarding decisional conflict (using the Decisional Conflict Scale DCS) and knowledge at baseline and after DA use three and six months post study inclusion. The subanalysis involved 236 women aged 18–40 and 181 women aged >40 years. At baseline, both age groups differed significantly in all socio-demographic variables, except *BRCA1/2* PV distributions. The younger age group displayed higher scores in the DCS subscale informed (*p* = .002) and higher knowledge (*p* = .010). Among the 18-40-year-olds, DA use (versus no DA) led to improvements in the DCS subscale informed at three (*p* = .025) and six months (*p* = .000). In the >40-year-olds, DA use (versus no DA) led to improvements in the DCS subscales informed (*p* = .028), values clarity (*p* = .028) and support (*p* = .030) and increased knowledge at three months (*p* = .048). These results indicate that both age groups benefited from DA use, but the older ones did so to a greater extent. This suggests that it might be useful to tailor DAs more closely to age- or life stage-related needs to enable more personalised care and support for women with *BRCA1/2* PVs.

## Introduction

Pathogenic variants (PVs) in the *BRCA1* and/or *BRCA2* (*BRCA1/*2) genes are associated with high risks of developing first and contralateral breast cancer (BC) and ovarian cancer (OC) [1]. Average lifetime risks are around 70% for first BC and around 44% (*BRCA1* PV) and 17% (*BRCA2* PV) for OC [1]. The average 20- to 25-year cumulative risks of contralateral BC range from about 25% to 34% (*BRCA2* PV) to 40% to 44% (*BRCA1* PV) [1–3]. Various preventive options are available for *BRCA1/2* PVs carriers to manage these risks. With regard to BC risks, intensified breast surveillance (and aftercare) (IBS/A), which can detect BC at an early, potentially curable stage [4], or risk-reducing mastectomy (RRM) are offered. Bilateral RRM in *BRCA1/2* PV carriers without previous cancer (previvors) results in a large reduction in BC risk [5] and may confer a survival benefit in *BRCA1* PV carriers [6]. Contralateral RRM in women after first BC (survivors) decreases BC morbidity and overall mortality [7], but is limited, since potential competing risks, such as a heightened BC recurrence risk, must be considered against the benefits of contralateral RRM. RRM poses an irreversible decision resulting in loss of natural breast and breastfeeding ability. It also requires downstream decisions such as the choice of breast reconstruction. Risk-reducing bilateral salpingo-oophorectomy (RRBSO) is the only effective preventive option so far to manage OC risk, and is usually recommended around the age of 40 [8]. RRBSO reduces OC risk, OC-specific and overall mortality [9, 10], which is highly important given the poor treatment options and prognosis of OC. However, it terminates fertility and can cause estrogenic-depleting side effects e.g., osteoporosis, cardiovascular disease, and vasomotor symptoms. These can be counteracted with hormone replacement therapy (HRT) up to the mean age when natural menopause would occur. Still, concerns about a possible increase in cancer risk with long-term use have not yet been resolved [11] and HRT is favoured for women without BC diagnosis [12].

As seen, each option coincides with advantages and disadvantages that substantially affect women’s lives, health, and well-being. Valuation and decision for an option can vary considerably depending on individual attitudes [13]. Several factors can impact the decision-making process. These include cognitive factors (e.g. own perception of risk and need for risk reduction [14], misunderstanding of information), personal factors (e.g. family and life planning considerations [15], family history and support), and psychological factors including psychopathology, body image and sexuality concerns [16–21]. These can trigger decisional conflicts that may lead to decision delay, decision regret or dissatisfaction [14, 15]. To reach the most suited decision, knowing and understanding of risks and options, clarity on the best type and timing of medical measure along with weighing up the consequences are necessary. Tools such as evidence-based decision aids (DAs) can assist PV carriers to make informed decisions based on evidence-based information and personal values and preferences [16].

Research has established DAs to effectively support the decision-making process for preventive strategies among these women [17], but there is a gap concerning potential differences depending on women’s age. However, individual age can also play an important role in decision-making, given that younger and older PV carriers usually face different risk, life circumstances and family situations. Younger women up to about 35–40 years have a lower average lifetime OC risk than those over 40 [1] and thus, objectively have more time to clarify their wants and needs, including reproductive wishes, and decide on their preventive measures. Yet, younger *BRCA1/2* PV carriers are often concerned about dating, family planning, reproduction, and career planning [18, 19] and feel increased pressure and urgency once they received the genetic test result [18–20]. Weighing up reproductive and breastfeeding wishes and risk-reducing surgery options often results in feelings of being overwhelmed [19, 21, 22]. In contrast, within older PV carriers, childbearing planning is mostly completed, and at their life stage, reasons in favour of RRBSO come to the fore, for example the desire to reduce cancer risk and achieve longer life expectancy [23]. Thus, older women may rather feel increased urgency due to their heightened risk status [19, 24] and concerns regarding surgical consequences and HRT following RRBSO [25, 26].

Therefore, it is essential to assess to what extent DAs can support women with *BRCA1/2* PVs at different age phases in their decision-making process, particularly women up to 40 and those >40 years. Recently, an evidence-based DA for women with *BRCA1/2* PVs was developed in two modifications (one for previvors, one for survivors) for the German context [27]. In a randomised controlled trial (RCT) with 417 women aged 18–68 (mean 39.8 ± 11.4) years, the DAs were shown to significantly reduce the scores for the decisional conflict scales total, informed, values clarity and support and to increase knowledge regarding BC/OC risks and preventive options, compared to no DA use [28, 29]. However, it remained unclear whether this pattern of results differs for women up to 40 versus those aged >40 years. The present exploratory subanalysis aimed to further investigate (i) the levels of decisional conflict and knowledge that *BRCA1/2* PV carriers aged 18–40 years versus those aged >40 years exhibited at baseline and (ii) the impact of DA use on these parameters within and between the two age groups. The findings could provide a more accurate insight into how DAs can support younger and older female *BRCA1/2* PV carriers and may offer indications of how future decision-making support interventions can be better tailored to the needs of different age groups to provide them with more personalised care and support.

## Materials and methods

### Study design and population

This descriptive exploratory subanalysis is derived from a broader prospective randomised controlled trial (RCT) that investigated the effectiveness of German decision aids (DAs) for women with *BRCA1/2* PVs [29]. The trial, conducted at the Centre for Familial Breast and Ovarian Cancer at the University Hospital Cologne, Germany, was approved by the Ethics Committee of the Faculty of Medicine of the University of Cologne, Germany [ethical approval dated 26 April 2017, reference number 17–128]. The original RCT was retrospectively registered on 14 June 2019 with the German Clinical Trials Register, DRKS-ID: DRKS0001523. The study’s protocol and methodology is published elsewhere [28]. In short, included were women with *BRCA1/2* PVs (previvors and survivors) who had a medical appointment for post-test genetic counselling (PTGC) or IBS/A. The RCT’s recruitment phase began on 17 January 2019 and ended on 6 October 2021. Prior to study start, all participants were informed about the study and had given written consent to take part in the study. Following informed written consent, the participants were randomly assigned to the intervention group (IG), obtaining usual care plus an evidence-based DA or to the control group (CG), obtaining usual care only. Previvors and survivors each received a DA modification tailored to their specific information needs [27]. After data collection at baseline (t0), the IG received the DA. Further data collections were three (t1) and six months (t2) post study inclusion. The present analysis was conducted with the sample of 417 participants who had completed the baseline questionnaire (t0). Women were categorised into the age groups 18–40 and >40 years. The cut-off at 40 years was chosen because this age phase marks a medically and personally relevant turning point for *BRCA1/2* PV carriers, particularly as decisions about completing family planning and risk-reducing adnexal surgeries [30] are necessary. Assessments were conducted for the younger and the older age group and for the IG and CG within each of the age groups.

### Decisional conflict and knowledge

Decisional conflict was measured with the Decisional Conflict Scale (DCS) [31, 32] comprising of 16 items. It encompasses a total scale and five subscales (informed, support, values clarity, uncertainty, and effective decision). Responses are rated on a five-point Likert scale (0 = *strongly agree* to 4 = *strongly disagree*). For each scale, the scores were calculated according to the manual [32]; a higher score reflects a higher conflict. Scores range from 0 (extremely low decisional conflict) to 100 (extremely high decisional conflict). Cronbach’s alpha for the DCS subscales ranged between 0.74 and 0.83. In this analysis, DCS total and the DCS subscales informed, values clarity, and support were assessed. Data was collected at baseline (t0), three months (t1) and six months (t2) post study inclusion.

Knowledge was assessed with an instrument consisting of 15 statements on risks and prevention options of BC/OC, targeted at previvors or survivors, respectively, so some of the statements (n = 6) were different. The instrument was developed by medical experts based on the counselling contents and action guidelines consented in Germany. The answer options were "*agree"*, "*disagree"* or "*don’t know"*. A total score was calculated based on the number of correct answers. It ranged from 0 to 15, with higher scores corresponding to a higher knowledge level. Cronbach’s alpha was 0.61. Data was collected at baseline (t0) and three months (t1) post study inclusion. More information on the knowledge scale is provided in S1 File.

### Statistical analyses

Baseline medical and demographic data were analysed for both the 18–40-year-old and >40-year-old groups, with comparisons made between the IG and the CG within each age group. Descriptive statistics were used to summarise the number of participants (n), mean scores, minimum and maximum scores, and standard deviations (SD). Differences in categorical variables were assessed using Fisher’s exact test, while differences in continuous variables were analysed using the two-sided independent t-test for normally distributed data; otherwise, the Mann-Whitney U-test was applied. Baseline comparisons between age groups 18–40 and >40 years included medical and demographic characteristics, DCS total and subscale scores, and knowledge scores. Additionally, differences between IG and CG within each age group were calculated at each time point for these variables. A significance level of .05 was applied for all analyses. Given the exploratory nature of this study, *p*-values between .05 and < .10 were considered marginally significant, indicating a notable trend. Benjamini-Hochberg adjustment was employed to correct for multiple testing. Analyses were conducted using IBM SPSS Statistics for Windows, Version 28.0. Armonk, NY: IBM Corp.

## Results

### Study population

Table 1 represents the baseline characteristics for the age groups 18–40 and >40 years regarding medical and demographic variables and baseline mean scores ± SD for DCS total, DCS subscales informed, values clarity and support and knowledge. There were 236 women in the 18–40 age group with a mean age of 31.3 ± 5.4 years and 181 women in the >40 age group with a mean age of 51.3 ± 6.1 years. Age groups did not differ in having *BRCA1*, *BRCA2* or both PVs (*p*_*adjusted*_ *=* .529), while all other medical and demographic characteristics differed significantly. The 18–40 age group was more likely to have had the genetic test results since under one year (53.8%) (*p*_*adjusted*_ *=* .010), be recruited at PTCG (42.8%) (*p*_*adjusted*_ = .002), have no cancer history (*p*_*adjusted*_ = .002), and have an academic educational status (*p*_*adjusted*_ = .002) compared to the >40 age group. Additionally, they were less likely to have children (*p*_*adjusted*_ = .002), have completed family planning (*p*_*adjusted*_ = .002), be in a partnership (*p*_*adjusted*_ = .002) and be employed (*p*_*adjusted*_ = .027) compared to the >40 age group. Regarding baseline decisional conflict and knowledge, women aged 18–40 years had statistically significantly higher mean scores for the DCS subscale informed (M: 37.1 ± 19.8) than women aged >40 years (M: 35.5 ± 22.9) (*p*_*adjusted*_ = .002). The other scores for the DCS subscales and the DCS total scale were non-significant between both age groups (*p* ≥ .05). The 18–40 age group displayed statistically significantly higher mean knowledge scores at baseline (M: 10.4 ± 2.7) compared to the >40 age group (M: 9.8 ± 2.6) (*p*_*adjusted*_ = .011).

**Table 1:** Baseline characteristics of the study population.

Baseline characteristic	Age group		Age group		p*
	18–40 years		>40 years		
	(n = 236)		(n = 181)		
	n	%	n	%	
**Medical**					
Pathogenic variant					.529
*BRCA1*^*a*^	137	58.1	96	53.0	
*BRCA2*	95	40.3	83	45.9	
*BRCA1* & *BRCA2*	4	1.7	2	1.1	
Recruitment at					
PTGC	101	42.8	39	21.5	**.002**
IBS/A	135	57.2	142	78.5	
Time since genetic test result					.**011**
≤ 1 year	120	53.8	69	38.8	
> 1 to ≤ 5 year	73	32.7	69	38.8	
> 5 years	30	13.5	40	22.5	
Cancer history					.**002**
No cancer history	186	78.8	104	57.5	
History of unilateral BC	48	20.3	77	41.6	
Children					**.002**
Yes	93	39.4	150	82.9	
No	142	60.2	30	16.6	
Completed family planning					.**002**
Yes	56	23.7	173	95.6	
No	155	65.7	4	2.2	
**Demographics**					
Mean age (years) [SD]	31.3	[5.4]	50.8	[6.6]	**.002**
Marital status					.**002**
Married/relationship	113	47.9	130	71.8	
Single	122	51.7	50	27.6	
Educational status					.**002**
Academic^b^	111	47.0	51	28.2	
Non-academic^c^	125	53.0	129	71.3	
Employment status					**.027**
Employed^d^	159	67.4	141	77.9	
Not employed^e^	76	32.2	39	21.5	
	**mean**	**SD**	**mean**	**SD**	p**
**Decisional conflict score**					
DCS total score	37.1	19.8	34.5	22.9	.129
DCS informed subscore	33.5	21.3	27.4	21.1	**.002**
DCS values clarity subscore	36.7	24.0	34.0	25.5	.221
DCS support subscore	26.2	19.1	28.5	19.9	.333
**Knowledge score**	10.5	2.7	9.8	2.6	**.010**

^a^one participant with a *BRCA1* PV also had a *CHEK2* PV;

^b^ includes: university degree, university of applied science degree;

^c^ includes: no degree, middle/intermediate school certification, final/technical high school certification;

^d^ includes: full/part time employment;

^e^ includes: school/training/studies, parental leave, unemployed, not able to work, retired, temporary job, not specified;

BC: breast cancer; *BRCA1/2*: breast cancer genes 1 and/or 2; DCS: Decisional Conflict Scale; PTGC: post-test genetic counselling; IBS/A: intensified breast surveillance (and aftercare); SD: standard deviation.

*B-H adjusted p-values calculated using Fisher’s exact test or independent t-test;

**B-H adjusted p-values calculated using two-sided Mann-Whitney-U-test

The IG versus CG were also compared in their baseline characteristics in each age group and results can be seen in S1 Table. There were no significant differences between IG and CG at baseline in either age group (*p* ≥ .05).

### Decisional conflict

**Fig 1:**
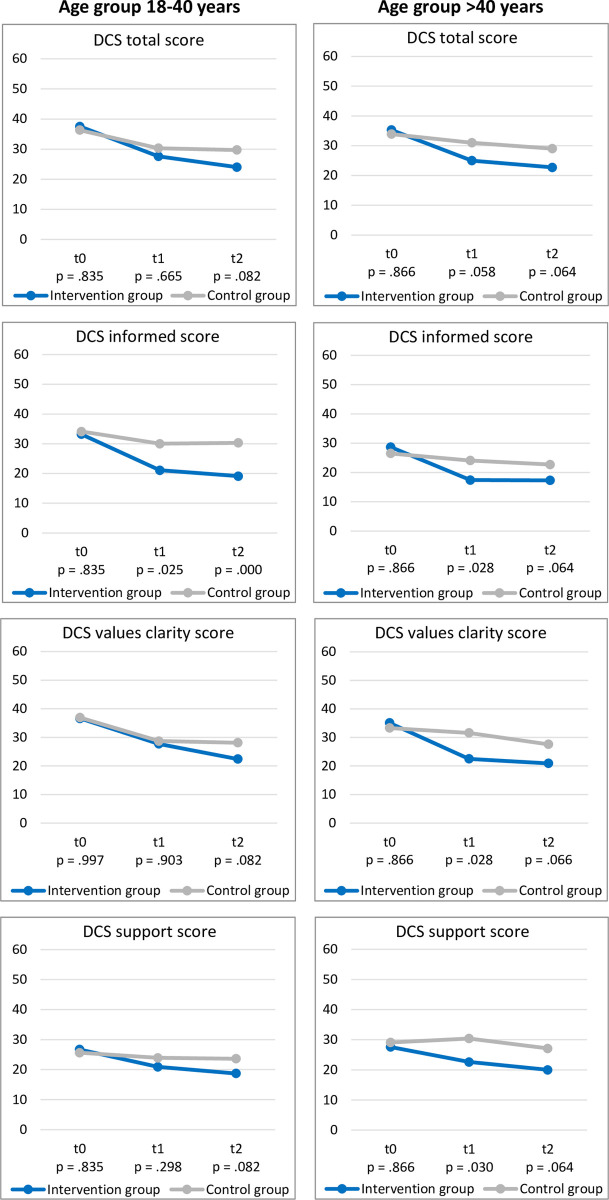
Decisional conflict scores according to age groups at baseline and after three and six months. Note: p-values reflect two-sided Mann-Whitney U tests between IG versus CG. All p-values are B-H-adjusted. DCS: Decional Conflict Scale.

Fig 1 displays the results for the DCS total score and the subscores informed, values clarity and support comparing IG versus CG within each of the age groups at baseline, three and six months post study inclusion.

Within the 18–40 age group, no differences between IG and CG were found at baseline (*p* ≥ .05). After three months, the IG (M: 22.1 ± 20.2) had significantly lower scores for the DCS subscale informed than the CG (M: 30.0 ± 21.7) (*p*_*adjusted*_ *=* .025). No other statistically significant differences were found (*p ≥* .05). After six months, the IG (M: 19.1 ± 17.8) still had statistically significantly lower scores for the DCS subscale informed than the CG (M: 30.3 ± 21.4) (*p*_*adjusted*_ *=* .000). Additionally, after six months, the IG displayed trends towards lower scores compared with CG for the DCS total scale (IG: M: 24.0 ± 19.0; CG: M: 29.7 ± 21.6) (*p*_*adjusted*_ *=* .082) and the subscales values clarity (IG: M: 22.4 ± 22.1; CG: M: 28.1 ± 24.7) (*p*_*adjusted*_ *=* .082) and support (IG: M: 18.7 ± 17.9; CG: M: 23.6 ± 19.9) (*p*_*adjusted*_ *=* .082).

Within the >40 age group, no differences between IG versus CG were found at baseline (*p* ≥ .05). After three months, the IG showed statistically significant lower scores for the DCS subscales informed (IG: M: 17.4 ± 18.2; CG: M: 24.1, SD = 18.9) (*p*_*adjusted*_ *=* .028), values clarity (IG: M = 22.5 ± 19.9; CG: M: 31.6 ± 23.1) (*p*_*adjusted*_ *=* .028) and support (IG: M: 22.6 ± 21.2; CG: M: 30.4 ± 21.6) (*p*_*adjusted*_ *=* .030) compared to CG. In addition, there was a noticeable trend in the DCS total scale, whereby the IG (M: 25.0 ± 19.4) showed marginally significantly lower scores compared to CG (M: 31.0 ± 20.7) (*p*_*adjusted*_ = .058). After six months, the IG displayed marked trends towards lower scores compared with CG for the DCS total scale (IG: M: 22.7 ± 18.3; CG: M: 29.0 ± 19.8) (*p*_*adjusted*_ = .064) and the subscales informed (IG: M: 17.3 ± 16.4; CG: M: 22.7 ± 17.6) (*p*_*adjusted*_ = .064), values clarity (IG: M: 20.9 ± 19.5; CG: M: 27.6 ± 23.0) (*p*_*adjusted*_ = .066) and support (IG: M: 20.0 ± 18.7; CG: M: 27.1 ± 20.0) *(p*_*adjusted*_ = .064). However, the differences fell marginally short of statistical significance. The underlying data of the descriptive statistics for the DCS total scores and all DCS subscores are listed in S2 Table.

### Knowledge

**Fig 2:**
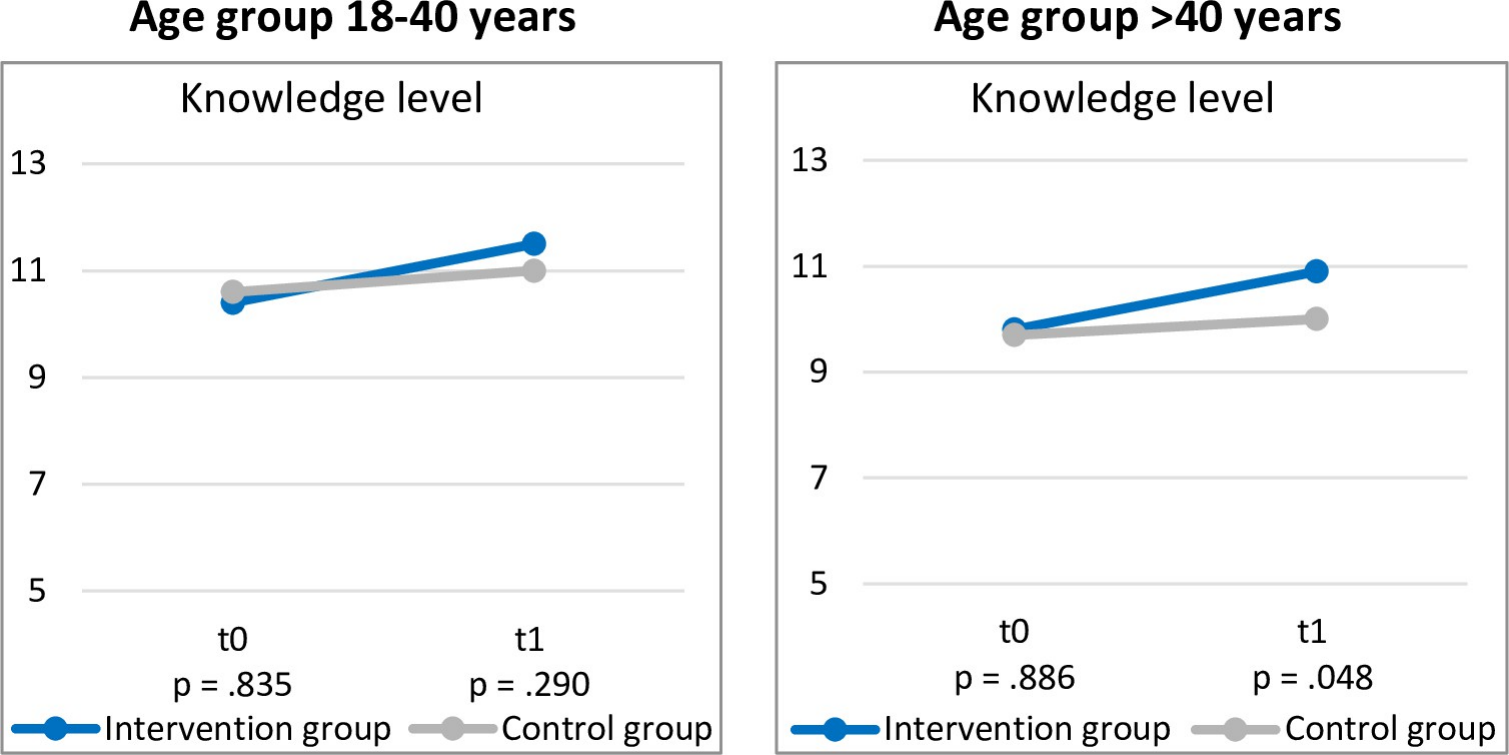
Knowledge scores according to age groups at baseline and after three months. Note: p-values reflect two-sided Mann-Whitney-U-test between IG versus CG. All p-values are B-H-adjusted.

Fig 2 shows the results for the knowledge scores comparing IG versus CG within both age groups at baseline (t0) and three months (t1) post study inclusion.

Comparing IG and CG within the 18–40 age group revealed no differences at baseline (IG: M: 10.4 ± 2.8; CG: M: 10.6 ± 2.5) (*p*_*adjusted*_ = .835) and three months post study inclusion (IG: M: 11.5 ± 2.8; CG: M: 11.0 ± 2.8) (*p*_*adjusted*_ = .290).

For the >40 age group, no difference in knowledge scores was found between IG (M: 9.8 ± 2.5) and CG (M: 9.7 ± 2.6) at baseline (*p*_*adjusted*_ = .866). However, three months post study inclusion, the IG (M: 10.9 ± 2.5) showed statistically significant higher knowledge scores than CG (M: 10.0 ± 2.7) (*p*_*adjusted*_ = .048). The underlying data of the descriptive statistics for the knowledge results are listed in S3 Table.

## Discussion

This study is based on an RCT which identified that women aged 18–68 years with *BRCA1/2* PVs presented significant improvements in decisional conflict and knowledge when receiving an evidence-based DA. The present subanalysis specifically examined the outcomes in the age groups 18–40 and >40 years to gain a better insight into how these age groups differed and whether the DA intervention had varying effects among these two groups.

### Study population

Baseline characteristics of the 18–40 and >40-year-olds showed that both age groups were comparable with respect of the distribution of *BRCA1/2* PVs, but significantly differed in all other medical and demographic parameters. Specifically, 18-40-year-olds were less likely to have had cancer, children or finished family planning and/or have a partner. This confirms that both age groups were in significantly different life and family planning phases, risk and health situations, or personal experiences with cancer, as described in previous research [19, 20].

Regarding baseline decisional conflict, both age groups exhibited comparable scores for all DCS scales except for the DCS subscale informed, where the 18-40-year-olds had a significantly higher score than women aged >40 years. This indicates that the younger women displayed higher decisional conflict due to feeling uninformed compared with the older age group. Interestingly, the younger age group displayed higher baseline knowledge compared to the >40 age group, suggesting a discrepancy between their objective knowledge level and their subjective feeling regarding their own knowledge level. This may suggest a conflict between the knowledge they acquired or received prior to the intervention and the knowledge and information needs deemed as necessary to make an informed decision. This aligns with research identifying that 63% of younger PV carriers report at least one unmet information need and a lack of access to information relevant for them [33]. This discrepancy may also be explained in that the younger PV carriers may have heightened information needs than those made available to them. Compared to the older age group, the younger PV carriers displayed a heightened prevalence of an academic degree. Research has shown a positive association between higher education status and increased information seeking [34] as well as increased *health* information seeking behaviour [35]. Therefore, it is possible that individuals with a higher education status have higher information needs and may tend to feel insufficiently informed. Correspondingly, several studies have discovered high information needs among younger *BRCA1/2* PV carriers [33, 36, 37]. Younger PV carriers expressed an increased wish for more detailed information about reproductive decisions [20] or weighing childbearing wishes against risk management options [19, 21]. Another explanation is that a more recent genetic test result and PTCG may lead to more unanswered questions and uncertainty in younger PV carriers, whilst older PV carriers with a less recent genetic test result and PTCG may feel more confidence in their knowledge due to having more time to process and research the topic. In contrast to the 18-40-year-olds, this study revealed that women aged >40 actually displayed lower baseline knowledge. This aligns with prior research identifying more misconceptions on several topics related to the PVs in older women [36]. They revealed more misunderstandings regarding the impact of a positive genetic test result on personal cancer progression (recurrence, metastases) and inheritance to offspring as well as expressed a greater need for lay-friendly and written information than younger PV carriers.

### Decisional conflict and knowledge after DA use

In line with the different information/knowledge needs, the DA intervention significantly increased knowledge levels in the >40-year-olds, but did not change in the 18-40-year-olds. The different response pattern may be explained by the initial higher educational status of the 18–40 age group at baseline, leading to a lack of knowledge improvement post-DA. Alternatively, the DA might not have been adequately tailored to the specific information needs of the 18–40 age group. The significant knowledge increase observed among those over 40 suggests they may have benefited substantially from the DA, possibly due to starting with lower baseline knowledge or the DA better addressing their information needs.

In both age groups, we observed a trend of decreased total decisional conflict scores, but with differing underlying reasons. Among the 18–40 age group, there was a statistically significant decrease in decisional conflict due to feeling uninformed in the IG versus CG after three and six months of DA use. This suggests that for younger PV carriers, DA use primarily led to a sustained decrease in decisional conflict resulting from self-perceived information deficits. Interestingly, this finding occurred despite no change in objective knowledge. It is possible that for younger women, the intervention made them realise the sufficiency and extent of their knowledge and consequently reduced their self-perceived deficits. However, further research is required to confirm this hypothesis. In contrast, within the IG of the >40 age group, the scores for decisional conflict due to feeling uninformed, unsupported and having poor values clarity had statistically significantly decreased after three months compared with the CG. This indicates that among older PV carriers, DA use caused a more comprehensive reduction in decisional conflict due to several underlying causes. The results support previous findings that DAs fundamentally improve decision-related outcomes in women with *BRCA1/2* PVs [17].

Interestingly, the older PV carriers experienced a much broader reduction of decisional conflict scores compared to CG after DA use than the younger ones. To the best of our knowledge, the effectiveness of DAs has not yet been specifically studied in *BRCA1/2* PV carriers aged >40 years. Therefore, the reasons for the present result can only be speculated but may be associated to some basic differences between the age groups: Compared to the younger women, twice as many of the older women had a BC history and in over 60% of cases and genetic test results were much longer in the past. This suggests that the >40-year-olds were more frequently confronted with cancer risks arising from *BRCA1/2* PVs combined with problems and worries of the previous BC diagnosis. Previous research has identified that women with PVs in moderate risk genes for hereditary BC and a BC history are more prone to misunderstandings regarding risk reduction or preventive information, complicating the decision-making process [38]. In addition, the older age group had received their medical consultation and information about their genetic test result and risk situation much longer ago and this alongside the lower educational level may result in lower knowledge levels. It is also plausible that older *BRCA1/2* PV carriers generally experience less decisional uncertainty due to the tendency of older age coinciding with a clearer life perspective, whereas younger age usually corresponds with a wider amount of life choices. Consequently, this may lead to older PV carriers having increased benefits in decisional conflict aspects after DA use, in particular regarding feeling better supported and perceiving more clarity in their values. This complex starting point, combined with the use of evidence-based DA modifications tailored to survivors’ information and decision-making needs [27, 29], could therefore contribute to a broader impactful decision- and knowledge-related support in the older age group.

Overall, this study extends on previous intervention studies by highlighting differences among *BRCA1/2* PV carriers aged 18–40 years and those >40 years at baseline and in the effectiveness of using a DA mainly targeted to a specific health decision. The results emphasise that it might be useful for DA developers to also tailor DAs more closely to needs associated with specific life phases. With such an approach, DAs might better target persons where they need information and support. Increasing support can promote the decision-making process as well as the decision quality. This can improve satisfaction with the decision made and be clinically relevant, as this can also positively influence patient’s health situation. Likewise, doctors who use or hand out DAs can benefit from the results in their daily practice. Being aware that DAs targeted to a specific health problem can address different age groups differently, can initiate a more targeted use of these tools in counselling practice.

Whilst various factors in addition to age such as the amount of information or emotional distress contribute to the decision-making process [39, 40], healthcare research is lacking a more personalised approach for *BRCA1/2* PV carriers. Generalised DAs fail to acknowledge individual differences such as age, education, health and life statuses and psychological wellbeing. Future research could explore personalised or intelligent DAs, as available for cancer patients: one personalised DA was developed specifically for men with prostate cancer which individualised outcomes based on the patients’ health state and personal preferences [41]. Additionally, in a recent scoping review on personalised decision support tools in the decision-making process of BC, various of interactive web-based clinical decision support tools were identified that were personalised, e. g. to tumour characteristics or age, yet there is a lack of research on the effectiveness, feasibility, and acceptability of these tools [40]. Further, Motorny et al. [39] note the absence of a theoretical framework or guidance for many of these web-based tools. Therefore, they developed a framework for future personalised decision aids considering four components: emotional adaptation, decision strategy, information needs and the application. The lack of theoretical framework in previous studies, alongside the findings of the present study highlighting age differences and pronounced individual group baseline differences underscore the need for further assessment of patient-centred and individualised interventions to improve decision-making for women with *BRCA1/2* PVs.

### Strengths and limitations

One notable strength of this study is its prospective RCT design, including a large number of participants. Notably, to the best of our knowledge, this was the first analysis to examine the impact of DA use on decisional conflict and knowledge levels in different age groups of women with *BRCA1/2* PVs. As the baseline results confirm that both the 18-40-year-groups and the >40-year-group showed a comparable distribution of *BRCA1/2* PVs, but differed in terms of all other medical and demographic characteristics, two well-defined groups with markedly different characteristics were compared. This provides a good basis to investigate possible age-related differences. However, it is important to consider the study’s limitations. Being an exploratory analysis, there is a question about the power of the study as the original trial was powered on the primary outcome. To overcome this and strengthen the study’s methodology, Benjamini-Hochberg corrections were used to correct for multiple testing. This may have contributed to the finding that DA use resulted in some clear trends in several outcomes compared to CG, yet these lacked statistical significance. Further research is required to fully clarify on the extent of these effects. Another limitation is the fact that the results are descriptive in nature and therefore no causal conclusions can be drawn. However, the data extend on the results of the underlying RCT and they indicate interesting differences in the baseline situation and the effects of DA in younger and older PV carriers. Further research is needed to identify possible underlying factors.

## Conclusion

The results of this analysis suggest that the use of evidence-based DAs can lead to differential outcomes dependent on age. In younger *BRCA1/2* PV carriers, DA use primarily improved decisional conflict related to self-perceived information deficits, although they already had a high knowledge level to begin with. In contrast, DA use in older *BRCA1/2* PV carriers improved decisional conflict related to feeling uninformed, having a poor values clarity, and feeling unsupported. In addition, the older group exhibited an increased knowledge level after DA use. This implies that DA use was able to promote important parameters of the decision-making process, whereby older PV carriers seemed to benefit to a greater extent than younger ones. As this analysis was conducted from an exploratory starting point, further research with a priori hypotheses is necessary to confirm the present findings and to further assess potential causal associations. Nevertheless, these findings may offer support for the need of patient-centred or personalised care and support for women with *BRCA1/2* PVs. Further research may benefit from investigating tailored decision support tools with specific age-related needs and other individual factors based on theoretical frameworks.

## Supporting information

S1 File Information on the knowledge scale used in the trial. (DOCX)

S1 Table Baseline characteristics of the study participants according to age groups including total, intervention and control groups. (DOCX)

S2 Table Descriptive statistics for DCS scores at t0, t1, and t2 for both age groups. (DOCX)

S3 Table Descriptive statistics for knowledge sum scores at t0 and t1 for both age groups. (DOCX)
