# Supplementary material for: Decisional conflict and knowledge in women with BRCA1/2 pathogenic variants: An exploratory age group analysis of a randomised controlled decision aid trial
Source: PLoS One. 2024 Oct 24;19(10):e0311432. doi: 10.1371/journal.pone.0311432 (PMC11500967; doi:10.1371/journal.pone.0311432)
Supplement: S1 File — (DOCX) [file pone.0311432.s001.docx]

**Information on the Knowledge Scale used in the trial**

Number of items:
The scale contains 15 statements about BC/OC risks and respective prevention options available in the German health care context. Each statement is to be rated as "agree", "disagree", or "don't know."
As *BRCA1/2* PV carriers without prior BC diagnosis (previvors) and those with prior BC diagnosis (survivors) differ in some aspects, they had to receive target group-specific statements. Therefore, some statement (n=6) differed.
For example: While the previvors had to evaluate statements about the risk of a first breast cancer, the survivors had to evaluate statements about the risk of a second breast cancer in the healthy breast.

Score building:
The number of correct answers was used to determine the knowledge level. For each participant, each correct answer was coded 1 and each incorrect answer (these included incorrect responses and the "don't know" response) was coded 0. Then, a knowledge sum score was formed. Sum score ranges from 0 (extremely low knowledge level) to 15 (extremely high knowledge level).

Information on scale development:
When the RCT was conducted, no scale was available that reflected the current research data on risks and preventive options for female *BRCA 1/2* PV carriers and that referred to the German S3-Guidelines. It was therefore necessary to develop a new knowledge scale to test knowledge level of the entire study population. Medical experts in the field of familial breast and ovarian cancer at the University Hospital Cologne developed the scale used in the RCT. The scale reflects essential contents that correspond to the guideline-based procedures in the German health context in the GC-HBOC centres and that are discussed with *BRCA1/2* PV carriers seeking advice as part of the genetic post-test counselling.

Prior to use in the RCT, the questionnaires t0, t2 and t3 (of which t0 and t1 contained the knowledge scale) were pre-tested for comprehensibility and adapted according to feedback.

Distribution of topics on cancer risks and preventive options:

- 4 items deal with the risks of BC/OC

- 4 items deal with risk-reducing breast surgery

- 3 items deal with

-- The intensified breast surveillance programme (for previvors)

-- The intensified breast surveillance and aftercare programme (for survivors)

- 3 items deal with preventive options for OC.

- 1 item addresses a topic that is explained in a section addressing ‘questions and answers’ of the DAs.

-- For previvors: breastfeeding, topic more asked by previvors

-- For survivors: balancing decision for RRCM with prognosis of the first BC, topic applies only to survivors

Internal consistency reliability:
Internal consistency reliability of the knowledge scale used in the RCT showed a Cronbach's alpha of 0.61. In line with previous research, this value was considered acceptable / moderate.

Abbreviations:
BC: breast cancer; *BRCA1/2:*  breast cancer genes 1 and/or 2; GC-HBOC: German Consortium for Hereditary Breast and Ovarian Cancer; OC: ovarian cancer; PV: pathogenic variant; RCT: randomized controlled trial; RRCM: risk-reducing contralateral mastectomy.
