# Supplementary material for: Decisional conflict and knowledge in women with BRCA1/2 pathogenic variants: An exploratory age group analysis of a randomised controlled decision aid trial
Source: PLoS One. 2024 Oct 24;19(10):e0311432. doi: 10.1371/journal.pone.0311432 (PMC11500967; doi:10.1371/journal.pone.0311432)
Supplement: S1 Table — (DOCX) [file pone.0311432.s002.docx]

**Supplemental Table 1: Baseline characteristics of the study population according to age groups including total, intervention and control groups**

|  |  |  | **Age group 18-40 years** | | | | |  |  | **Age group >40 years** | | | | |
| --- | --- | --- | --- | --- | --- | --- | --- | --- | --- | --- | --- | --- | --- | --- |
| **Baseline characteristic** | **Total group**  **n=236** | | **IG (n=139)** | | **CG (n=97)** | | ***p**** | **Total group**  **n=181** | | **IG (n=77)** | | **CG (n=104)** | | ***p**** |
|  | **n** | **%** | **n** | **%** | **n** | **%** |  |  |  | **n** | **%** | **n** | **%** |  |
| ***Medical*** |  |  |  |  |  |  |  |  |  |  |  |  |  |  |
| Pathogenic variant |  |  |  |  |  |  | .734 |  |  |  |  |  |  | .723 |
| *BRCA1^a^* | 137 | 58.1 | 78 | 56.1 | 59 | 60.8 |  | 96 | 53.0 | 46 | 59.7 | 50 | 48.1 |  |
| *BRCA2* | 95 | 40.3 | 58 | 41.7 | 37 | 38.1 |  | 83 | 45.9 | 30 | 39.0 | 53 | 51.0 |  |
| *BRCA1* & *BRCA2* | 4 | 1.7 | 3 | 2.2 | 1 | 1.0 |  | 2 | 1.1 | 1 | 1.3 | 1 | 1.0 |  |
| Recruitment |  |  |  |  |  |  |  |  |  |  |  |  |  |  |
| PTGC | 101 | 42.8 | 62 | 44.6 | 39 | 40.2 | .734 | 39 | 21.5 | 19 | 24.7 | 20 | 19.2 | .723 |
| IBS/A | 135 | 57.2 | 77 | 55.4 | 58 | 59.8 |  | 142 | 78.5 | 58 | 75.3 | 84 | 80.8 |  |
| Time since genetic test result |  |  |  |  |  |  | .734 |  |  |  |  |  |  | .723 |
| ≤ 1 year | 120 | 53.8 | 75 | 54.0 | 45 | 46.4 |  | 69 | 38.8 | 31 | 40.3 | 38 | 36.5 |  |
| > 1 to ≤ 5 years | 73 | 32.7 | 42 | 30.2 | 31 | 32.0 |  | 69 | 38.8 | 27 | 35.1 | 42 | 40.4 |  |
| > 5 years | 30 | 13.5 | 15 | 10.8 | 15 | 15.5 |  | 40 | 22.5 | 19 | 24.7 | 21 | 20.2 |  |
| Cancer history |  |  |  |  |  |  | .734 |  |  |  |  |  |  | .723 |
| No cancer history | 186 | 78.8 | 112 | 80.6 | 74 | 76.3 |  | 104 | 57.5 | 45 | 58.4 | 59 | 56.7 |  |
| History of unilateral BC | 48 | 20.3 | 27 | 19.4 | 21 | 21.6 |  | 77 | 41.6 | 32 | 41.6 | 45 | 58.4 |  |
| Children |  |  |  |  |  |  | .30 |  |  |  |  |  |  | .30 |
| Yes | 93 | 39.4 | 63 | 45.3 | 30 | 30.9 |  | 150 | 82.9 | 58 | 75.3 | 92 | 88.5 |  |
| No | 142 | 60.2 | 76 | 54.7 | 66 | 68.0 |  | 30 | 16.6 | 18 | 23.4 | 12 | 11.5 |  |
| Completed family planning |  |  |  |  |  |  | .890 |  |  |  |  |  |  | .723 |
| Yes | 56 | 23.7 | 33 | 23.7 | 23 | 23.7 |  | 173 | 95.6 | 70 | 90.9 | 103 | 99.0 |  |
| No | 155 | 65.7 | 93 | 66.9 | 62 | 63.9 |  | 4 | 2.2 | 3 | 3.9 | 1 | 1.0 |  |
| ***Demographics*** |  |  |  |  |  |  |  |  |  |  |  |  |  |  |
| Mean age (years) [SD] | 31.3 | [5.4] | 31.0 | [5.7] | 31.7 | [5.0] | .734 | 50.8 | [6.6] | 51.3 | [6.1] | 50.5 | [7.0] | .723 |
| Marital status |  |  |  |  |  |  | .734 |  |  |  |  |  |  | .723 |
| Married/relationship | 113 | 47.9 | 69 | 49.6 | 44 | 45.4 |  | 130 | 71.8 | 54 | 70.1 | 76 | 73.1 |  |
| Single | 122 | 51.7 | 70 | 50.4 | 52 | 53.6 |  | 50 | 27.6 | 23 | 29.9 | 27 | 26.0 |  |
| Educational status |  |  |  |  |  |  | .734 |  |  |  |  |  |  | .780 |
| Academic^b^ | 111 | 47.0 | 61 | 43.9 | 50 | 51.5 |  | 51 | 28.2 | 21 | 27.3 | 30 | 28.8 |  |
| Non-academic^c^ | 125 | 53.0 | 78 | 56.1 | 47 | 48.5 |  | 129 | 71.3 | 56 | 72.7 | 73 | 70.2 |  |
| Employment status |  |  |  |  |  |  | .450 |  |  |  |  |  |  | .723 |
| Employed^d^ | 159 | 67.4 | 88 | 63.3 | 71 | 73.2 |  | 141 | 77.9 | 63 | 81.8 | 78 | 75.0 |  |
| Not employed^e^ | 76 | 32.2 | 51 | 36.7 | 25 | 25.8 |  | 39 | 21.5 | 14 | 18.2 | 25 | 24.0 |  |

^a^one participant with a *BRCA1* PV also had a CHEK2 PV; ^b^includes: university degree, university of applied science degree; ^c^includes: no degree, middle/intermediate school certification, final/technical high school certification; ^d^includes: full/part time employment; ^e^includes: school/training/studies, parental leave, unemployed, not able to work, retired, temporary job, not specified; BC: breast cancer; BRCA1/2: breast cancer genes 1 and/or 2; PTGC: post-test genetic counselling; IBS/A: intensified breast surveillance (and aftercare); IG: intervention group; CG: control group; SD: standard deviation.
*B-H adjusted p-values reflect differences between IG and CG conducted with Fisher’s exact or independent t-tests
