# Supplementary material for: Decisional conflict and knowledge in women with BRCA1/2 pathogenic variants: An exploratory age group analysis of a randomised controlled decision aid trial
Source: PLoS One. 2024 Oct 24;19(10):e0311432. doi: 10.1371/journal.pone.0311432 (PMC11500967; doi:10.1371/journal.pone.0311432)
Supplement: S2 Table — (DOCX) [file pone.0311432.s003.docx]

**S2 Table.**

**Descriptive Statistics for DCS Scores at t0, t1, and t2 for both age groups**

**Age group 18-40 years**

| **Baseline (t0)** | **Age group 18-40 years** | | | | | | | | | | | | | | |  |
| --- | --- | --- | --- | --- | --- | --- | --- | --- | --- | --- | --- | --- | --- | --- | --- | --- |
|  | Total group | | | | | Intervention group | | | | | Control group | | | | |  |
| DCS Score | n | min | max | mean | SD | n | min | max | mean | SD | n | min | max | mean | SD | *p-*value* |
| DCS Informed | 235 | 0.0 | 100.0 | 33.5 | 21.3 | 139 | 0.0 | 100.0 | 33.2 | 22.3 | 96 | 0.0 | 75.0 | 34.1 | 19.7 | .835 |
| DCS Values Clarity | 232 | 0.0 | 100.0 | 36.7 | 24.0 | 137 | 0.0 | 83.3 | 36.6 | 22.8 | 95 | 0.0 | 100.0 | 36.9 | 25.7 | .997 |
| DCS Support | 236 | 0.0 | 75.0 | 26.2 | 19.1 | 139 | 0.0 | 75.0 | 26.7 | 19.5 | 97 | 0.0 | 75.0 | 25.6 | 18.6 | .835 |
| DCS Uncertainty | 234 | 0.0 | 100.0 | 51.1 | 28.4 | 139 | 0.0 | 100.0 | 51.7 | 28.0 | 95 | 0.0 | 100.0 | 50.1 | 29.1 | .702 |
| DCS Effective Decision | 231 | 0.0 | 100.0 | 37.5 | 25.9 | 138 | 0.0 | 100.0 | 38.5 | 25.4 | 93 | 0.0 | 100.0 | 36.0 | 26.7 | .400 |
| DCS Total | 225 | 0.0 | 84.4 | 37.1 | 19.8 | 137 | 0.0 | 78.1 | 37.5 | 19.7 | 88 | 0.0 | 84.4 | 36.3 | 20.0 | .835 |

**p-values reflect two-sided Mann-Whitney U tests between IG versus CG; all p-values are B-H-adjusted. DCS: Decional Conflict Scale*

| **After 3 months (t1)** | **Age group 18-40 years** | | | | | | | | | | | | | | |  |
| --- | --- | --- | --- | --- | --- | --- | --- | --- | --- | --- | --- | --- | --- | --- | --- | --- |
|  | Total group | | | | | Intervention group | | | | | Control group | | | | |  |
| DCS Score | n | min | max | mean | SD | n | min | max | mean | SD | n | min | max | mean | SD | *p-*value* |
| DCS Informed | 221 | 0.0 | 83.3 | 25.4 | 21.2 | 128 | 0.0 | 83.3 | 22.1 | 20.2 | 93 | 0.0 | 75.0 | 30.0 | 21.7 | .**025** |
| DCS Values Clarity | 222 | 0.0 | 100.0 | 28.1 | 24.5 | 129 | 0.0 | 91.7 | 27.7 | 23.7 | 93 | 0.0 | 100.0 | 28.7 | 25.7 | .903 |
| DCS Support | 223 | 0.0 | 75.0 | 22.2 | 19.7 | 130 | 0.0 | 66.7 | 20.9 | 19.7 | 93 | 0.0 | 75.0 | 23.9 | 19.7 | .298 |
| DCS Uncertainty | 223 | 0.0 | 100.0 | 41.6 | 28.9 | 130 | 0.0 | 100.0 | 41.9 | 27.9 | 93 | 0.0 | 100.0 | 41.0 | 30.3 | .654 |
| DCS Effective Decision | 215 | 0.0 | 100.0 | 29.1 | 24.2 | 124 | 0.0 | 100.0 | 28.7 | 23.0 | 91 | 0.0 | 100.0 | 29.6 | 25.8 | .989 |
| DCS Total | 213 | 0.0 | 87.5 | 28.7 | 20.4 | 122 | 0.0 | 76.6 | 27.6 | 19.2 | 91 | 0.0 | 87.5 | 30.3 | 22.0 | .665 |

**p-values reflect two-sided Mann-Whitney U tests between IG versus CG; all p-values are B-H-adjusted. DCS: Decional Conflict Scale*

| **After 6 months (t2)** | **Age group 18-40 years** | | | | | | | | | | | | | | |  |
| --- | --- | --- | --- | --- | --- | --- | --- | --- | --- | --- | --- | --- | --- | --- | --- | --- |
|  | Total group | | | | | Intervention group | | | | | Control group | | | | |  |
| DCS Score | n | min | max | mean | SD | n | min | max | mean | SD | n | min | max | mean | SD | *p-*value* |
| DCS Informed | 218 | 0.0 | 83.3 | 23.8 | 20.1 | 126 | 0.0 | 75.0 | 19.1 | 17.8 | 92 | 0.0 | 83.3 | 30.3 | 21.4 | **.000** |
| DCS Values Clarity | 218 | 0.0 | 91.7 | 24.8 | 23.3 | 126 | 0.0 | 91.7 | 22.4 | 22.1 | 92 | 0.0 | 91.7 | 28.1 | 24.7 | .082 |
| DCS Support | 217 | 0.0 | 75.0 | 20.7 | 18.9 | 125 | 0.0 | 66.7 | 18.7 | 17.9 | 92 | 0.0 | 75.0 | 23.6 | 19.9 | .082 |
| DCS Uncertainty | 218 | 0.0 | 100.0 | 37.2 | 28.6 | 126 | 0.0 | 100.0 | 36.2 | 28.3 | 92 | 0.0 | 100.0 | 38.5 | 29.0 | .552 |
| DCS Effective Decision | 216 | 0.0 | 100.0 | 26.3 | 24.2 | 125 | 0.0 | 100.0 | 24.1 | 23.0 | 91 | 0.0 | 100.0 | 29.3 | 25.5 | .141 |
| DCS Total | 215 | 0.0 | 78.1 | 26.4 | 20.3 | 124 | 0.0 | 78.1 | 24.0 | 19.0 | 91 | 0.0 | 76.6 | 29.7 | 21.6 | .082 |

**p-values reflect two-sided Mann-Whitney U tests between IG versus CG; all p-values are B-H-adjusted. DCS: Decional Conflict Scale*

**Age group > 40 years**

| **Baseline (t0)** | **Age group > 40 years** | | | | | | | | | | | | | | |  |
| --- | --- | --- | --- | --- | --- | --- | --- | --- | --- | --- | --- | --- | --- | --- | --- | --- |
|  | Total group | | | | | Intervention group | | | | | Control group | | | | |  |
| DCS Score | n | min | max | mean | SD | n | min | max | mean | SD | n | min | max | mean | SD | *p-*value* |
| DCS Informed | 180 | 0.0 | 91.7 | 27.4 | 21.1 | 77 | 0.0 | 91.7 | 28.7 | 21.3 | 103 | 0.0 | 91.7 | 26.5 | 21.0 | .866 |
| DCS Values Clarity | 180 | 0.0 | 100.0 | 34.0 | 25.5 | 76 | 0.0 | 100.0 | 35.1 | 27.1 | 104 | 0.0 | 100.0 | 33.3 | 24.3 | .866 |
| DCS Support | 178 | 0.0 | 83.3 | 28.5 | 19.9 | 76 | 0.0 | 83.3 | 27.6 | 21.4 | 102 | 0.0 | 83.3 | 29.1 | 18.8 | .866 |
| DCS Uncertainty | 179 | 0.0 | 100.0 | 47.6 | 29.2 | 77 | 0.0 | 100.0 | 47.4 | 31.2 | 102 | 0.0 | 100.0 | 47.8 | 27.8 | .817 |
| DCS Effective Decision | 178 | 0.0 | 100.0 | 34.2 | 27.0 | 76 | 0.0 | 100.0 | 36.4 | 29.5 | 102 | 0.0 | 100.0 | 32.6 | 24.9 | .484 |
| DCS Total | 171 | 0.0 | 95.3 | 34.5 | 22.0 | 74 | 0.0 | 95.3 | 35.3 | 24.0 | 97 | 0.0 | 87.5 | 33.9 | 20.4 | .866 |

**p-values reflect two-sided Mann-Whitney U tests between IG versus CG; all p-values are B-H-adjusted. DCS: Decional Conflict Scale*

| **After 3 months (t1)** |  | | **Age group > 40 years** | | | | | | | | | | | | | |  |
| --- | --- | --- | --- | --- | --- | --- | --- | --- | --- | --- | --- | --- | --- | --- | --- | --- | --- |
|  | Total group | | | | | | Intervention group | | | | |  |  | Control group | | |  |
| DCS Score | n | min | | max | mean | SD | n | min | max | mean | SD | n | min | max | mean | SD | *p-*value* |
| DCS Informed | 170 | 0.0 | | 83.3 | 21.3 | 18.8 | 72 | 0.0 | 66.7 | 17.4 | 18.2 | 98 | 0.0 | 83.3 | 24.1 | 18.9 | **.028** |
| DCS Values Clarity | 170 | 0.0 | | 100.0 | 27.7 | 22.2 | 72 | 0.0 | 75.0 | 22.5 | 19.9 | 98 | 0.0 | 100.0 | 31.6 | 23.1 | **.028** |
| DCS Support | 168 | 0.0 | | 100.0 | 27.0 | 21.7 | 72 | 0.0 | 83.3 | 22.6 | 21.2 | 96 | 0.0 | 100.0 | 30.4 | 21.6 | **.030** |
| DCS Uncertainty | 171 | 0.0 | | 100.0 | 39.4 | 28.5 | 72 | 0.0 | 100.0 | 35.9 | 28.2 | 99 | 0.0 | 100.0 | 41.9 | 28.5 | .145 |
| DCS Effective Decision | 168 | 0.0 | | 100.0 | 28.2 | 23.4 | 71 | 0.0 | 87.5 | 27.0 | 22.5 | 97 | 0.0 | 100.0 | 29.0 | 24.2 | .663 |
| DCS Total | 162 | 0.0 | | 87.5 | 28.4 | 20.3 | 71 | 0.0 | 73.4 | 25.0 | 19.4 | 91 | 0.0 | 87.5 | 31.0 | 20.7 | .058 |

**p-values reflect two-sided Mann-Whitney U tests between IG versus CG; all p-values are B-H-adjusted. DCS: Decional Conflict Scale*

| **After 6 months (t2)** | **Age group > 40 years** | | | | | | | | | | | | | | |  |
| --- | --- | --- | --- | --- | --- | --- | --- | --- | --- | --- | --- | --- | --- | --- | --- | --- |
|  | Total group | | | | | Intervention group | | | | |  | Control group | | | |  |
| DCS Score | n | min | max | mean | SD | n | min | max | mean | SD | n | min | max | mean | SD | *p-*value* |
| DCS Informed | 164 | 0.0 | 66.7 | 20.5 | 17.3 | 67 | 0.0 | 66.7 | 17.3 | 16.4 | 97 | 0.0 | 66.7 | 22.7 | 17.6 | .064 |
| DCS Values Clarity | 162 | 0.0 | 75.0 | 24.8 | 21.9 | 67 | 0.0 | 75.0 | 20.9 | 19.5 | 95 | 0.0 | 75.0 | 27.6 | 23.0 | .066 |
| DCS Support | 164 | 0.0 | 83.3 | 24.2 | 19.8 | 67 | 0.0 | 75.0 | 20.0 | 18.7 | 97 | 0.0 | 83.3 | 27.1 | 20.0 | .064 |
| DCS Uncertainty | 163 | 0.0 | 100.0 | 35.5 | 26.7 | 67 | 0.0 | 91.7 | 32.2 | 25.4 | 96 | 0.0 | 100.0 | 37.8 | 27.5 | .145 |
| DCS Effective Decision | 161 | 0.0 | 100.0 | 25.5 | 22.0 | 66 | 0.0 | 75.0 | 23.3 | 21.1 | 95 | 0.0 | 100.0 | 27.0 | 22.6 | .277 |
| DCS Total | 158 | 0.0 | 81.3 | 26.4 | 19.4 | 66 | 0.0 | 71.9 | 22.7 | 18.3 | 92 | 0.0 | 81.3 | 29.0 | 19.8 | .064 |

**p-values reflect two-sided Mann-Whitney U tests between IG versus CG; all p-values are B-H-adjusted. DCS: Decional Conflict Scale*
