# Supplementary material for: Decisional conflict and knowledge in women with BRCA1/2 pathogenic variants: An exploratory age group analysis of a randomised controlled decision aid trial
Source: PLoS One. 2024 Oct 24;19(10):e0311432. doi: 10.1371/journal.pone.0311432 (PMC11500967; doi:10.1371/journal.pone.0311432)
Supplement: S3 Table — (DOCX) [file pone.0311432.s004.docx]

**S3 Table.**

**Descriptive Statistics for Knowledge sum scores at t0 and t1 for both age groups**

**Age group 18-40 years**

|  | **Age group 18 - 40 years** | | | | | | | | | | | | | | |  |
| --- | --- | --- | --- | --- | --- | --- | --- | --- | --- | --- | --- | --- | --- | --- | --- | --- |
|  | Total group | | | | | Intervention group | | | | | Control group | | | | |  |
| Knowledge sum score | n | min | max | mean | SD | n | min | max | mean | SD | n | min | max | mean | SD | *p-*value* |
| At baseline (t0) | 236 | 2.0 | 15.0 | 10.5 | 2.7 | 139 | 4.0 | 15.0 | 10.4 | 2.8 | 97 | 2.0 | 15.0 | 10.6 | 2.5 | .835 |
| After 3 months (t1) | 226 | 1.0 | 15.0 | 11.3 | 2.8 | 133 | 3.0 | 15.0 | 11.5 | 2.8 | 93 | 1.0 | 15.0 | 11.0 | 2.8 | .290 |

**p-values reflect two-sided Mann-Whitney U tests between IG versus CG; all p-values are B-H-adjusted.*

**Age group > 40 years**

|  | **Age group > 40 years** | | | | | | | | | | | | | | | |  |
| --- | --- | --- | --- | --- | --- | --- | --- | --- | --- | --- | --- | --- | --- | --- | --- | --- | --- |
|  | Total group | | | | | Intervention group | | | | | Control group | | | | | |  |
| Knowledge sum score | n | min | max | mean | SD | n | min | max | mean | SD | n | min | max | mean | SD | *p-*value* | |
| At baseline (t0) | 180 | 2.0 | 15.0 | 9.8 | 2.6 | 76 | 2.0 | 14.0 | 9.8 | 2.5 | 104 | 2.0 | 15.0 | 9.7 | 2.6 | .866 | |
| After 3 months (t1) | 170 | 2.0 | 15.0 | 10.4 | 2.6 | 72 | 4.0 | 15.0 | 10.9 | 2.5 | 98 | 2.0 | 15.0 | 10.0 | 2.7 | **.048** | |

**p-values reflect two-sided Mann-Whitney U tests between IG versus CG; all p-values are B-H-adjusted.*

**Age group 18-40 years versus age group > 40 years**

|  | **Age group 18 – 40 years**  **(total: IG & CG)** | | | | | **Age group > 40 years**  **(total: IG & CG)** | | | | |  |
| --- | --- | --- | --- | --- | --- | --- | --- | --- | --- | --- | --- |
| Knowledge sum score | n | min | max | mean | SD | n | min | max | mean | SD | *p-*value* |
| At baseline (t0) | 236 | 2,0 | 15,0 | 10,5 | 2,7 | 180 | 2,0 | 15,0 | 9,8 | 2,6 | **.007** |
| After 3 months (t1) | 226 | 1,0 | 15,0 | 11,3 | 2,8 | 170 | 2,0 | 15,0 | 10,4 | 2,6 | **.000** |

**p-values reflect two-sided Mann-Whitney U tests between both age groups; all p-values are B-H-adjusted.*
